# Colonic adenocarcinoma with synchronous metastasis to the pancreas: A case report and literature review

**DOI:** 10.1002/jgh3.12731

**Published:** 2022-03-28

**Authors:** Jennifer Yoon, Arpine Petrosyan, Timothy Wang, Adnan Ameer

**Affiliations:** ^1^ Department of Internal Medicine University of California San Francisco California USA; ^2^ Department of Gastroenterology and Hepatology University of California San Francisco California USA

**Keywords:** cancer, clinical practice and treatment, colorectal, diagnosis and therapy, pancreas

## Abstract

Metastatic lesions to the pancreas are a rare entity and make up about 0.5–5% of all pancreatic malignancies. Synchronous pancreatic metastasis is even less frequently reported. Before the widespread use of advanced endoscopic techniques, distinguishing between primary and secondary malignancies of the pancreas was diagnostically challenging. The accuracy of diagnosing metastatic lesions to the pancreas using endoscopic ultrasound with fine needle aspiration is around 91%. Distinguishing between primary and secondary lesions is crucial in determining disease management. We present a case of a young man who presented with synchronous pancreatic metastasis from colon adenocarcinoma.

## Graphical abstract

Metastatic lesions to the pancreas are a rare entity and make up about 0.5 to 5% of all pancreatic malignancies. Distinguishing between primary and secondary lesions is crucial in determining disease management. We present a case of a young man who presented with synchronous pancreatic metastasis from colon adenocarcinoma.

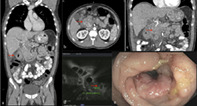

## Introduction

The most common sites for colorectal cancer (CRC) metastasis are regional lymph nodes, liver, bone, lungs, and peritoneum.^1^ Pancreatic metastasis from colon cancer is rare, and cases of synchronous pancreatic metastasis are practically nonexistent in the literature and limited to case reports. To the best of our knowledge, there have only been five reported cases of synchronous pancreatic metastasis from colon cancer, all cases occurring in patients in their 60s and 70s. We present a unique case of a 33‐year‐old male with right upper quadrant (RUQ) pain subsequently diagnosed with colon adenocarcinoma with synchronous metastasis to the pancreas.

## Case report

A 33‐year‐old male with a history of alcohol and tobacco abuse was transferred from an outside facility after presenting with intractable abdominal pain. He reported intermittent RUQ pain that started a few months ago along with a 35‐lb weight loss of unclear time frame. He denied any vomiting, constipation, hematochezia, melena, or hematemesis. Physical exam was positive for a palpable firmness in the RUQ with overlying tenderness and negative for jaundice. Laboratory results were significant for aspartate aminotransferase 107 U/L (8–40 U/L), alanine aminotransferase 192 U/L (10–40 U/L), alkaline phosphate 806 U/L (25–100 U/L), total bilirubin 2.6 mg/dl (0.3–1.2 mg/dl), direct bilirubin 1.7 mg/dl (0–0.4 mg/dl), alpha fetoprotein <1.3, and markedly elevated carcinoembryonic antigen (CEA) of 287.4 ng/ml (0–5.0 ng/ml) and carbohydrate antigen 19‐9 (CA‐19–9) of 148.2 U/ml (0–35 U/ml).

**Figure 1:**
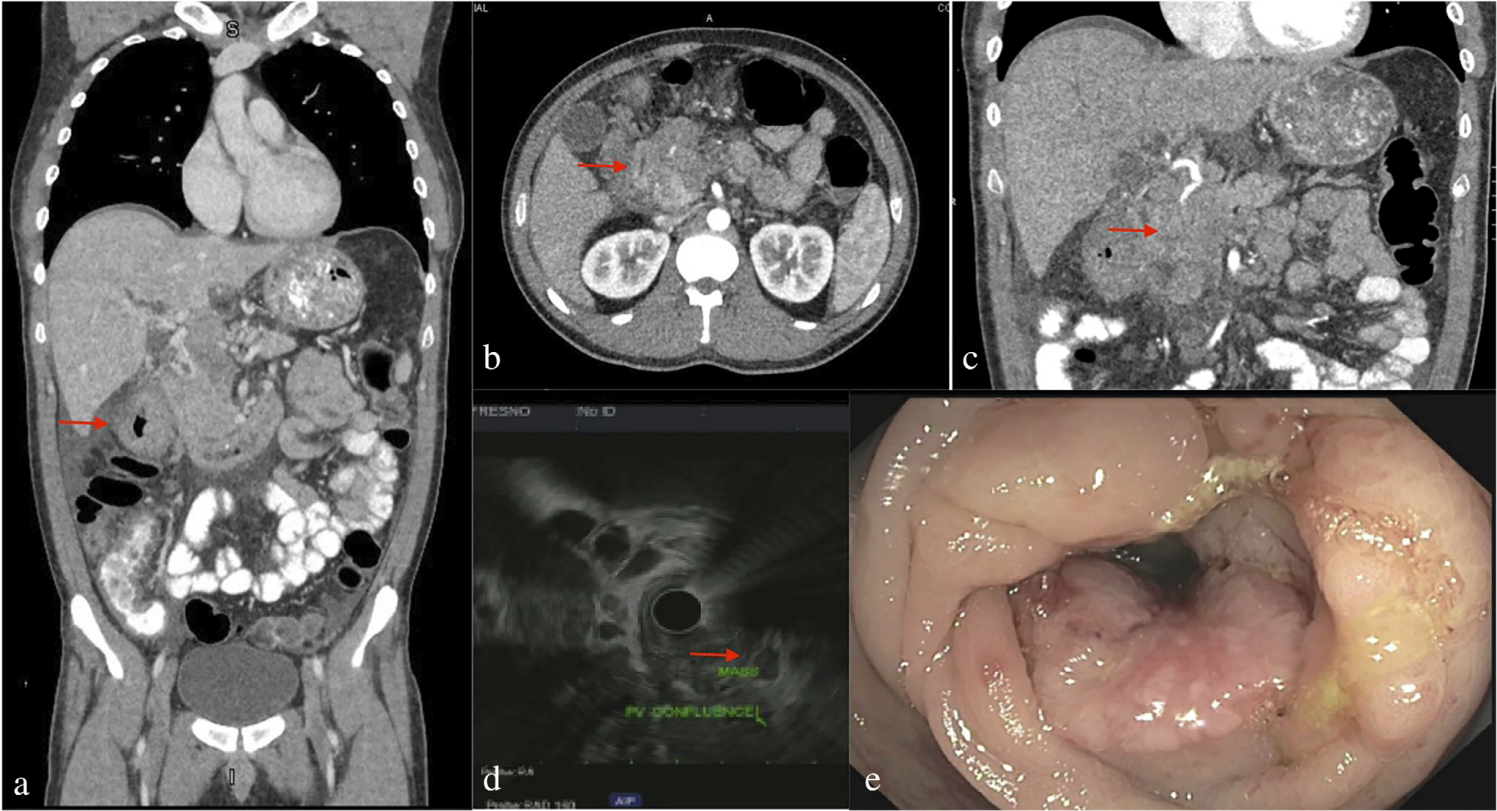
(a) Computed tomography (CT) scan showing large apple‐core, non‐obstructive mass in proximal transverse colon. (b, c) CT scan demonstrating pancreatic head mass. (d) Endoscopic ultrasound showed irregular, hypoechoic, heterogeneous lesion with portal vein invasion. Staged T3N2MX. (e) Colonoscopy showing partially obstructing mass with abnormal, ulcerated mucosa in the proximal transverse colon.

Computed tomography (CT) with pancreatic protocol showed a 4.4 × 5.4 × 7.6 cm pancreatic head mass with dilation of the intrahepatic biliary tree and pancreatic duct, occlusion of the superior mesenteric vein (SMV), numerous peripancreatic lymph nodes, two hepatic masses, and a large apple‐core, non‐obstructive mass of the proximal transverse colon measuring 10 cm in length (Fig. 1a–c). Magnetic resonance cholangiopancreatography (MRCP) similarly demonstrated mild dilations of the central intrahepatic, common bile duct, and main pancreatic duct to the level of pancreatic head. Endoscopic retrograde cholangiopancreatography (ERCP) was performed, which showed a malignant‐looking biliary stricture in the lower third of the main bile duct. A plastic biliary stent was placed. Endoscopic ultrasound (EUS) with fine needle aspiration (FNA) revealed an irregular, hypoechoic, heterogenous mass in the pancreatic head measuring 4.5 cm × 5.0 cm with loss of interface with the SMV–portal vein confluence (Fig. 1d). This was staged T3N2MX by endosonographic criteria. The mass was biopsied and pathology suggested adenocarcinoma, with immunohistochemical (IHC) staining supporting colonic primary (positive CK20, CDX2, and CD138). Given the invasion of the portal vein and SMV, hepatobiliary surgery did not recommend metastasectomy. Subsequently, a colonoscopy identified a partially obstructing tumor involving the distal ascending colon, hepatic flexure, and proximal transverse colon with pathology consistent with moderately differentiated adenocarcinoma (Fig. 1e).

He was discharged and underwent palliative chemotherapy. Unfortunately, interval imaging showed worsening disease with local lymph node metastasis. The patient was subsequently admitted for acute cholangitis in the setting of malignant obstruction complicated by Klebsiella bacteremia and septic shock, and he ultimately died 10 months after his initial diagnosis.

## Discussion

CRC is the third leading cause of cancer deaths in the United States. Metastasis occurs via the lymphatics, direct invasion, and along blood vessels, with the most common sites being regional lymph nodes, liver, bone, lungs, and peritoneum.^1^ This mechanism of spread makes metastatic lesions to the pancreas (PMETs) of colorectal origin highly unusual. In general, pancreatic metastasis from distant sites are not commonly seen. Reported frequency of metastatic disease to the pancreas vary between 0.5 and 5% of all pancreatic malignancies.^2^, ^3^ PMETs are typically discovered 1–3 years after initial diagnosis of CRC but have been reported to occur as far out as 10 years. They can be detected during initial work‐up for staging, on surveillance imaging, or during evaluation for symptoms. While 45–71% of PMETs from CRC are asymptomatic, some can present with abdominal pain and obstructive jaundice when the mass is in the head of the pancreas.^3^, ^4^ Most pancreatic metastases are initially discovered on abdominal CT with a sensitivity of 68–86% and specificity of 50–64%.^5^ Several patterns of occurrence have been described but the most common is a solitary lesion often with a hypodense focus from tumor necrosis.^6^

Prior to the utilization of EUS/FNA, solitary pancreatic lesions were a diagnostic challenge due to a lack of specificity between primary and secondary lesions on imaging. FNA sensitivities for metastatic disease range from 75% to 95% and specificity from 60 to 100%, with accuracy greater than 91%.^7^, ^8^ On EUS, metastatic lesions to the pancreas are typically reported as hypoechoic heterogeneous masses with well‐defined margins, with the head of the pancreas being the most common site.^2^, ^7^, ^8^ Other findings favoring secondary lesions include multiple lesions, a lack of a retention cyst, main pancreatic duct dilation, and pancreatic atrophy.^8^

Treatment options for all pancreatic metastasis are determined by the primary pathology, genetic markers and mutations, stage, and tumor burden. Treatment of pancreatic metastasis of colorectal primary consists of chemotherapy and more recently metastatecomies of solitary lesions, which are recommended on an individual basis.^2^, ^3^ It is difficult to comment on the prognosis and survival time of synchronous pancreatic metastasis because of a paucity of data. The reported 5‐year survival rate in colon cancer with distant metastasis is approximately 14%, and metastatic spread to more than one distant organ is associated with worse survival outcomes.^9^

We presented a unique case of synchronous pancreatic metastasis from colon cancer in a young, previously healthy patient. While solitary pancreatic lesions are usually a primary process, metastatic disease should always be considered especially when there is a question of two different primary malignancies. As demonstrated by our case, although extremely rare, colonic adenocarcinoma can metastasize to the pancreas. Therefore, if synchronous pancreatic metastasis is suspected, the colon should be evaluated. Prompt and accurate diagnosis is imperative for appropriate treatment planning given that treatment can be radically different for two primary malignancies and advanced colon cancer.
